# Trends and patterns of family planning methods used among women attending family planning clinic in a rural setting in sub-Sahara Africa: the case of Mbalmayo District Hospital, Cameroon

**DOI:** 10.1186/s13104-018-3658-1

**Published:** 2018-08-02

**Authors:** Paul Nkemtendong Tolefac, Theophile Njamen Nana, Eugene Vernyuy Yeika, Nkemnji Stanley Awungafac, Yolande Ntsama, Phillipe Nana Njotang

**Affiliations:** 1 Mbalmayo District Hospital, Mbalmayo, Cameroon; 2 Faculty of Medicine and Biomedical Sciences, University of Yaoundé 1, Yaoundé, Cameroon; 3 Faculty of Health Sciences, University of Buea, Buea, Cameroon; 4 Bangante District Hospital, Bangante, Cameroon

**Keywords:** Contraceptive, Family planning, Clinic, Birth control, Unwanted pregnancy

## Abstract

**Objective:**

Family planning enables women to prevent unwanted pregnancies and control family sizes. Provision of family planning services is an essential human right. This study aimed to describe the trends and patterns of contraceptive use in a family planning clinic in a rural district hospital setting.

**Results:**

A total 313 participants who used contraceptives between March 2016 and August 2017 were included this study given a. Their mean age was 32.4 ± 1.8 years with an age range of 18–48 years. The index study estimates the rate of contraceptive use at 17.4 contraceptives per month. The most commonly used contraceptive methods were implants and IUD in 29.4 and 28.4% of the participants respectively while the least used was condoms in 8.3% of the participants. Contraceptive used are highest among those 21–40 years (83.1%) and least among adolescents less than 20 years (6.7%).

## Introduction

Family planning (FP) refers to the provision of methods or services used to prevent pregnancies and enable families to space out children [1, 2]. This allows women to have control over their own health and have children when they are ready [3]. Family planning and contraceptive methods are key issues in reproductive health [4]. According to reports of the international conference on population and development that held in Cairo in 1994, reproductive health includes the right of men and women to be informed and to have access to safe, effective, affordable, and acceptable methods of FP of their choice for regulation of fertility [1, 5].

In the past 40 years, FP services have played a major role in raising the prevalence of contraceptive practice from less than 10–60% and reducing fertility in developing countries from about six to three births per woman [1]. However, in low middle income countries (LMICs) mainly in sub-Saharan Africa, contraceptive practice remains low while fertility, population growth, and unmet need for family planning are high [1].

According to the World Health Organisation recent statistics, an estimated 214 million women in developing countries would like to delay or stop childbearing but are not using any form of contraception [2]. Other benefits of family planning apart from spacing or stopping child bearing include preventing pregnancy-related health risks in women, reducing infant mortality, helping to prevent human immunodeficiency virus/acquired immune deficiency syndrome, empowering people and enhancing education, reducing adolescent pregnancies, and slowing population growth [2, 3]. Globally, contraceptive used has increased in many parts of the world but remained low in sub-Sahara Africa [3]. In Cameroon and according to the 2011 demographic and health survey (DHS), the contraceptive prevalence among women aged 15–49 years is 14% with a wide variations among regions with some regions such as the far north having very slow rates such as 3% and regions such as centre having slightly higher rates (25%) [6]. This same DHS in 2011 estimated the fertility rate at 5.1% [6]. A recent study conducted in Yaoundé, Cameroon, estimates the prevalence of unmet need of family planning/contraception at 20.4% [7].

The family planning clinic of the maternity service, Mbalmayo District Hospital (MDH), provides a complete suite of sexual and reproductive health services since 2016: intrauterine devices (IUDs), implants (levonorgestrel [LNG]), depot medroxy progesterone acetate (DMPA) also called Depo-Provera, combined oral contraceptives (COC), progesterone-only contraceptives (POP), emergency contraceptive pills, and condoms. Given that few studies have evaluated the contraceptive choices in Cameroon [4, 7–11] and no study has evaluated family planning methods in Mbalmayo since the inception of the family clinic in 2016, we conducted this study to review the contraception practice among patients attending the FP clinic of MDH. The study aimed specifically to determine the rate of contraceptive use and describe the trends and pattern of contraceptive use in this FP clinic.

## Main text

### Methods

#### Study design and settings

We conducted a descriptive cross sectional study at the FP clinic of the maternity service in Mbalmayo District Hospital (MDH). We retrospectively reviewed medical files of the patients who have used any FP method at the FP clinic from March 1, 2016 to August 31, 2017 (18 months). We included all patients aged greater than or equal to 18 years who received FP services in the FP clinic of MDH and we excluded all minors (< 18 years) and files with incomplete information. MDH is a 4th category hospital (district hospital) located in Mbalmayo health district in the centre region of Cameroon. The FP clinic of MDH is located in the maternity. The clinic operate daily consulting and counselling patients with needs for FP services. It has a focal point for FP who is a nurse with masters in reproductive health. Patients are first seen by a doctor who consults with and counsels them on different FP options. Then they are referred to a nurse who provides them with the FP method they have chosen.

#### Data collection and statistical analysis

The minimum sample size was calculated using the Lorenz formula, ${\text{N}} = \frac{{{\text{p}}\left( {1 - {\text{p}}} \right){\text{Z}}_{\text{crit}}^{2} }}{{{\text{d}}^{2} }}$ [12], where N is the minimum sample size, p = national prevalence of modern contraceptive use in Cameroon which is 14% [6], Z_crit_ is estimated from the Fisher and Yates tables at 1.96, d is the standard normal variate otherwise the minimum standard error to be tolerated by the researcher which is 0.05 for a 95% confidence interval. Putting these values in the equation and computing gives a minimum sample size of 185 participants.

Data was collected from medical records using case report form. We collected sociodemographic, medical and obstetric information as well as information on the type of contraceptive using case report form. This data was entered into Epi Info version 3.5.3 and then exported to SPSS version 20.0 for analysis. We determined mean and standard deviations of continuous variables and frequency and percentage of categorical variables.

#### Ethical considerations

Written informed consent was obtained from all participants. Ethical clearance was obtained from Mbalmayo District Hospital. We respected all the principles of ethics involving human participants according to the Helshinki declarations.

### Results

#### Baseline sociodemographic, obstetric and medical characteristics

A total of three hundred and thirteen (313) women benefitted from family planning using modern contraceptive methods between March 2016 and August 2017 in the FP clinic of MDH. There were all females with an age range of 18–48 years, mean age of 32.4 ± 1.8 years and most of our participants 20–39 years as seen on Table 1. This gives a contraceptive rate of 208.6 contraceptives per annum or 17.4/month.

**Table 1:** Baseline sociodemographic, obstetrics and medical characteristics of the participants

Characteristic	Category	Frequency	Percentage
Age	< 20	21	6.7
	20–29	128	40.9
	30–39	132	42.2
	≥ 40	32	10.2
Marital status	Single	32	52.4
	Married	164	31.6
	Divorce	99	10.2
	Widow	18	5.8
Profession	Civil servant	21	6.7
	Private sector	56	17.9
	House wife	45	14.4
	Student	89	28.4
	Unemployed	102	32.6
Level of education	No formal education	33	10.5
	Primary	99	31.6
	Secondary	103	32.9
	University	78	24.9
	No pregnancy	12	3.8
Gravid status	1 pregnancy	126	40.3
	2–4 pregnancies	99	31.6
	5–9 pregnancies	64	20.4
	> 10 pregnancies	12	3.8
Cardiovascular risk factors	Hypertension	8	2.6
	Diabetes	5	1.6
	Smoking	12	3.8
	Chronic kidney diseases	2	0.6

**Fig. 1:**
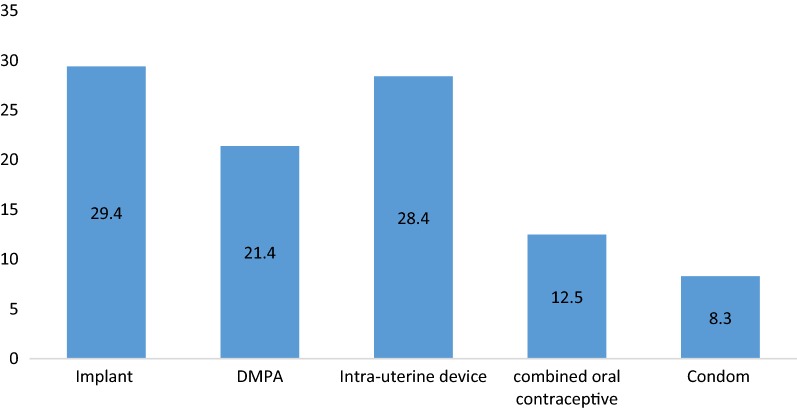
Patterns of different family planning methods used

As shown on Fig. 1 above, about 4/5th of the participants were between 20 and 39 years and about 1/3rd of the participants were married. The table further shows that all the participants have had at least one pregnancy prior to contraception.

#### Trends and patterns of family planning methods

**Fig. 2:**
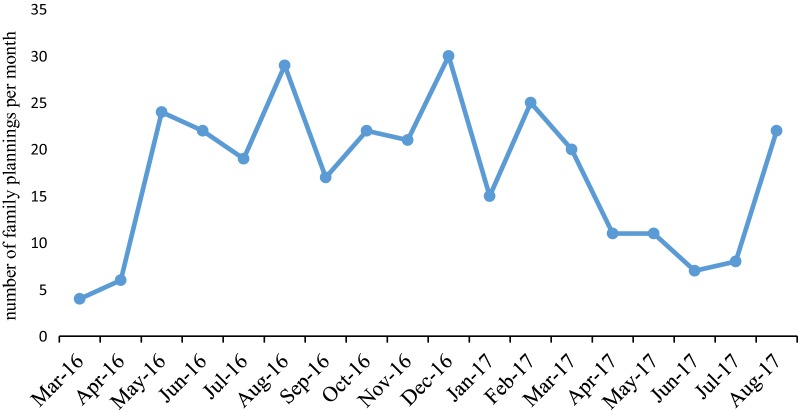
Trends in the utilisation of family planning over 18 months period between March 2016 and August 2017

The number of women who received contraception were estimated at 17.4/month. Three most common types of contraceptives used in descending order were: implants, intrauterine device (IUD), and 3 monthly injectable depot medroxy progesterone acetate (DMPA) (Fig. 1). As shown on Fig. 1, 29.4% of the participants chose implants, 28.4% chose intrauterine device and 21.4% chose depot medroxy progesterone acetate (DMPA) The utilization of contraceptive methods did not follow any particular pattern (Fig. 2).

### Discussion

The current study described the patterns and trends of contraceptive use in a district hospital in Cameroon. There were 313 participants all females with an age range of 15–48 years and a mean age of 32.4 ± 1.8 years. The index study estimates rate of contraceptive use at 17.4 contraceptives per month. The most commonly used FP methods were implants and IUD in 29.4 and 28.4% of the participants respectively while the least used was condoms in 8.3% of the participants. The absence of male participants could be explained by the fact that FP is conceived by Cameroonians as concerning women coupled to the fact the FP clinic is located in the maternity widely attended by females [4]. Similar results with females predominant in FP have been gotten elsewhere in Cameroon [4] and Nigeria [3]. A recent study by Njamen et al. [13] in Douala Cameroon on acceptability of vasectomy demonstrated a low acceptability rate of vasectomy, a form of male contraception, by men influenced by sociocultural and religious barriers.

The age of the participants ranges from 15 to 48 years with a mean age of 32.4 ± 1.8 years and most of the participants 20–39 years. This age group is mostly made up of working class sexually active women who want to avoid pregnancy and be professionally productive. Similar age ranges have been recently described in large urban areas in Cameroon [4] and Nigeria [3]. Adolescents (less than 20 years) were the least represented group with 6.7% of participants. The low rate of contraceptive use among adolescents is similar to findings of the Cameroonian 2011 DHS [6]. Similar low rate of contraceptive used among adolescents was earlier described in Yaoundé, a large FP centre by Yangsi et al. [4]. The direct implication of low rate of contraceptive use amongst adolescents is that these they presents with unwanted pregnancies and criminal clandestine abortions. About a third of the participants in this study were married (164, 31.6%). This can be explained by the fact that married women are more inclined to seek family planning methods with the motivation to limit family sizes. Similar trends have been observed in studies elsewhere in Africa [3, 10, 14, 15]. The motivation to do so might be based on the ever-increasing challenges of raising children and the need to limit their family sizes. A large proportion of the participants in this study have had at least one child (94.2%), with 40.3% having 2–4 children. This is similar to that obtained in Nigeria [3], Ghana [16] and Ethiopia [17]. Cameroonian DHS 2011 found contraceptive use to be highest among women with at least one child [6]. This can further be explained by the fact that women do not begin to use contraception until they have had at least one child and they become increasingly aware of the need to prevent unwanted pregnancies and limit family sizes after the first pregnancy. The proportion of participants with at least a university education (24.9%) was far lower than that obtained in Lagos Nigeria by Okunade et al. (77.6%) [3], but similar to that obtained in Osun state Nigeria (23.8%) [15]. This suggests that less educated women were seen in this study because of its rural setting which is similar to Osun state Nigeria unlike the metropolitan Lagos city that had more educated women. The relationship between contraceptive use and female education has been demonstrated in previous studies [18].

In this current study, the most commonly used FP methods were implants in 29.4% of the participants and IUDs in 28.4% of the participants whereas the least used methods were condoms in 8.3% of the participants. Surgical methods such as bilateral tubal ligation and vasectomy as well as diaphragms and progesterone only contraceptives were not used in the FP clinic of MDH during the study period. This is similar to majority of the studies in Nigeria [3, 19] and Cameroon [4] where IUDs and implants are among the most commonly used methods of contraception. This can be explained by the increasing preference for longer, reversible and more reliable methods of contraception by women.

### Conclusions

The rate of contraceptive used remain relatively low in the family planning clinic of Mbalmayo District Hospital. The most commonly used methods of contraceptives are implants and IUDs. Relatively few of the study participants (few of the clinic patients) who received contraception were adolescents.

## Limitations

Though our study provide important information on the patterns and use of contraceptives in a rural setting, it however has the following limitations:

- Poor record keeping system currently being used in the family planning clinic, which affected the accurate data collection as some information such as indications for contraceptive usage as well as complications of the contraceptives were not captured in the medical records.
- The study was hospital-based and not community-based and as such the findings may not be applicable to the general population.
- Contractive methods received were considered used. While this is true for some methods such as implants, IUDs and injectable DMPA, it may not be true for methods such as OCPs and condoms.

We therefore recommend continuous counselling and education of especially adolescents on the important of contraceptives in the prevention of unwanted pregnancies.

## Abbreviations

FP: family planning
MDH: Mbalmayo District Hospital
DMPA: depot medroxy progesterone acetate
DHS: Demographic Health Survey
